# A segmentation network for farmland ridge based on encoder-decoder architecture in combined with strip pooling module and ASPP

**DOI:** 10.3389/fpls.2024.1328075

**Published:** 2024-02-01

**Authors:** Qingqing Hong, Yue Zhu, Wei Liu, Tianyu Ren, Changrong Shi, Zhixin Lu, Yunqin Yang, Ruiting Deng, Jing Qian, Changwei Tan

**Affiliations:** ^1^ Jiangsu Key Laboratory of Crop Genetics and Physiology, Agricultural College of Yangzhou University, Yangzhou, China; ^2^ Jiangsu Key Laboratory of Crop Cultivation and Physiology, Agricultural College of Yangzhou University, Yangzhou, China; ^3^ Jiangsu Co-Innovation Center for Modern Production Technology of Grain Crops, Joint International Research Laboratory of Agriculture and Agri-Product Safety of the Ministry of Education of China, Jiangsu Province Engineering Research Center of Knowledge Management and Intelligent Service, College of Information Engineer, Yangzhou University, Yangzhou, China

**Keywords:** remote sensing, semantic segmentation, farmland ridge, strip pooling, encoder-decoder

## Abstract

In order to effectively support wheat breeding, farmland ridge segmentation can be used to visualize the size and spacing of a wheat field. At the same time, accurate ridge information collecting can deliver useful data support for farmland management. However, in the farming ridge segmentation scenarios based on remote sensing photos, the commonly used semantic segmentation methods tend to overlook the ridge edges and ridge strip features, which impair the segmentation effect. In order to efficiently collect ridge information, this paper proposes a segmentation method based on encoder-decoder of network with strip pooling module and ASPP module. First, in order to extract context information for multi-scale features, ASPP module are integrated in the deepest feature map. Second, the remote dependence of the ridge features is improved in both horizontal and vertical directions by using the strip pooling module. The final segmentation map is generated by fusing the boundary features and semantic features using an encoder and decoder architecture. As a result, the accuracy of the proposed method in the validation set is 98.0% and mIoU is 94.6%. The results of the experiments demonstrate that the method suggested in this paper can precisely segment the ridge information, as well as its value in obtaining data on the distribution of farmland and its potential for practical application.

## 1 Introduction

One of the most fundamental uses of remote sensing data in the field of agriculture management is mapping and monitoring farmland information. Field ridges are used in farming information to divide farmland into several crop zones and assist farmers in planning and managing their crops logically (Li et al., 2020; S. Wang et al., 2023). In wheat breeding, the division of ridges can help control the spread of pests and diseases and cross-contamination (Jiaguo et al., 2023), and the reasonable distribution of ridges can help provide crops with appropriate moisture and temperature to improve crop yield and quality (Zhang et al., 2023). Therefore, reliably and effectively extracting farmland ridge information from low-altitude remote sensing data is crucial for farmland management and decision-making.

More and more researchers have been utilizing remote sensing photos to carry out in-depth research on the distribution of farmland in recent years. When working with remote sensing images, the process of manually drawing farmland distribution information is easily influenced by subjective variables, and the data sources are dispersed, which makes it difficult to meet the demands of effective farmland management. The development of machine learning enables the automatic segmentation of farming data (Adebiyi et al., 2020; Kilwenge et al., 2021; Ibrahim Mohammad Abuzanouneh et al., 2022) and provides the corresponding algorithm support for remote sensing picture processing. For instance, to address the issue of similar objects having different spectra, (Xiao et al., 2019) utilized the CART decision tree classification algorithm and produced a spatial distribution map of farmland based on the spectral similarity between picture pixels. A stratified object-based farmland extraction method based on image region division was also proposed by (Xu et al., 2019) at the same time. To divide up farmland in remote sensing images with high spatial resolution, the image region was divided using the grey level co-occurrence matrix method over the whole image, and scale segmentation parameters were computed in local regions. The concept of regional division was also employed by (Cai et al., 2022). In the study of extracting cropland parcels, the image was first broadly segmented into several regions, and then the final cropland parcels were finely segmented based on average local variance function. In order to automatically segment and extract selected farmland regions, (Li et al., 2019) proposed an edge-preserving smoothing method to automatically segment and extract selected farmland regions, which segments and extracts farmland information with different features from remote sensing images based on the features of the ideally smoothed image, and maintains the boundaries of the farmland regions by using a maximum a posteriori estimation model. In order to overcome the effects of unstructured environments like uneven illumination, shadows and weather, (Liu et al., 2016) converted the original color image to grayscale and minimized the intuitionistic fuzzy divergence to obtain the ideal threshold for detecting various types of obstacles in segmented farmland.

However, the implementation of the above methods relies more on the similarity of pixels, and lacks the extraction of spatial and texture features of high-resolution images, resulting in limited accuracy of obtained farmland information. With the rapid development of deep learning, convolutional neural networks have been able to extract rich semantic information, (Hamano et al., 2023; J. Wang et al., 2023; Punithavathi et al., 2023) thereby alleviating the above deficiencies. (Masoud et al., 2020) designed a multiple dilation fully convolutional network to detect boundaries of agricultural fields and achieve farmland segmentation. To achieve pixel-by-pixel segmentation using a full convolutional network, however, takes a lot of time and more processing resources during training. As a result, some lightweight CNN models that perform well and have fewer parameters have drawn a lot of interest. Through the use of the spatial attention module and the channel attention module, respectively, (Cao et al., 2023) based on the Mask R-CNN network and combined with the feature pyramid of the dual attention mechanism, realized the automatic division of small farm farmland. In order to improve the detection of the edge region in the task of segmenting farmland, (Huan et al., 2022) proposed a multiple attention encoder–decoder network, designed a dual-pooling efficient channel attention module, and added a global-guidance information upsample module to the decoder. To more accurately capture the detailed information and boundary information in farmland segmentation, (Shunying et al., 2023) created a boundary-semantic-fusion deep convolution network. This network fused the boundary features and semantic features together and retained the spatial details and boundary information in the features. Despite this, when completing the task of field segmentation, it is important to take into account the unique characteristics of the vast span and narrow shape of the ridge. Therefore, (Zhang et al., 2021) created the strip pooling module and the mixed pooling module in conjunction with strip pooling in their study of ridge and farmland vacancy segmentation, which can capture the shape features and edge information of ridges well. However, the model is unable to obtain rich contextual semantic information when extracting the high-level semantic features using downsampling due to the limitation of the receptive field, which affects the connectivity of segmentation.

To address the above problems, this paper develops a segmentation method based on encoder-decoder architecture of strip pooling and atrous spatial pyramid pooling module (ASPP) to realize the segmentation of ridge information in crop fields using high-resolution farmland remote sensing images as a dataset. In this study, the model is referred as ASPNet for short. By comparing with several other existing semantic segmentation models, the model achieves the best results in accuracy and mean Intersection over Union (mIoU), and the output ridge shapes have good connectivity. The main contributions of this paper are as follows:

1. An encoder-decoder architecture is utilized because of the intricacy and irregularity of ridge edges. The architecture performs feature fusion during upsampling and gradually restores the feature map to the original feature map resolution size. The shallow edge texture features are preserved during the layer-by-layer feature fusion process.

2. Strip pooling is added to the standard decoder in response to the ridge’s slender and narrow shape. During the fusion process, it can enhance the feature map’s long-distance dependence in both the vertical and horizontal directions and capture the ridge’s strip-like shape characteristics.

3. ASPP architecture is added at the end of the encoder since the ridge information in remote sensing images involves a variety of widely fields. Different receptive fields are constructed by atrous convolution with different sampling rates to enhance the correlation of global spatial information, thus improving the connectivity of the ridge segmentation effect.

The rest of this paper is organized as follows. The materials and methods are described in Section 2 of this paper. Section 3 of this paper presents the results of experimental. The discussion of the experimental results is presented in Section 4. Finally, Section 5 gives conclusions and suggestions for future work.

## 2 Materials and methods

**Figure 1:**
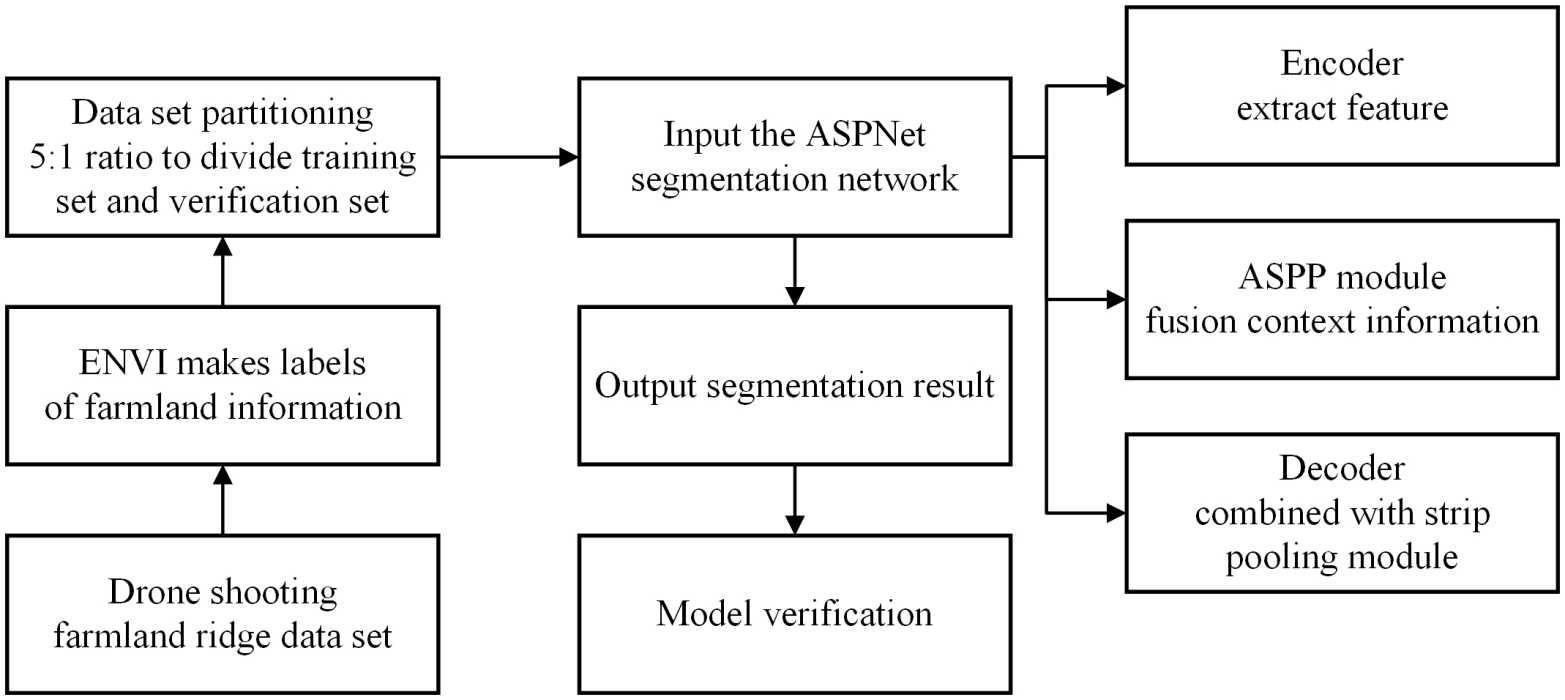
Research process flow chart.

As shown in **Figure 1**. This is the flowchart of the whole study. The methodology consists of three main phases: ridge dataset collection and processing, model design and model validation. Detailed descriptions of these steps are given in sections 2.1-2.3.

### 2.1 Data collection

**Figure 2:**
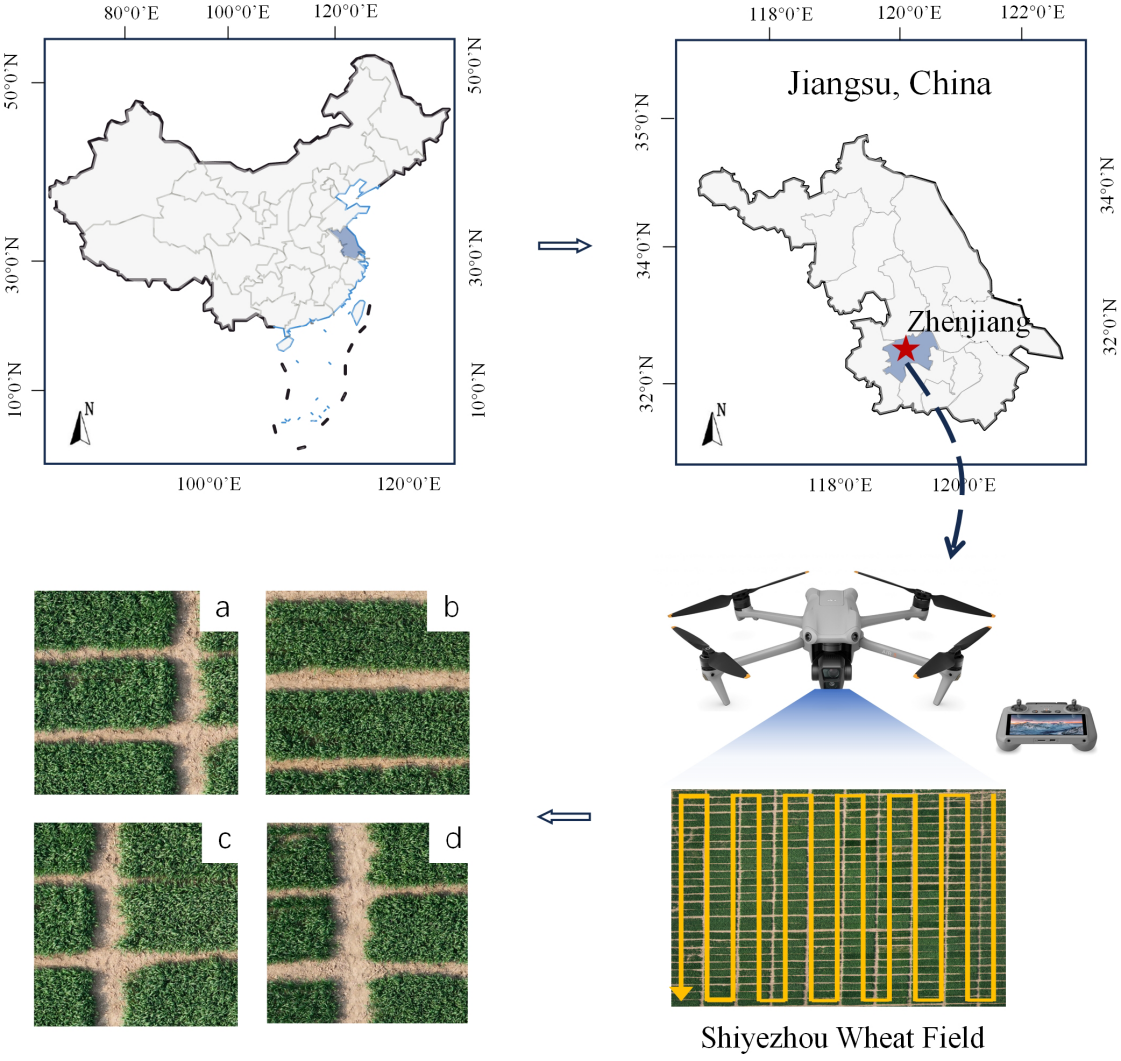
Data collection information.

The image data were taken in March 2023 at Shiyezhou, Zhenjiang, Jiangsu Province, China, covering an area of 7,063 square meters (shown in **Figure 2**). The shooting location was Yangzhou University Wheat - Zhenjiang Dantu Experimental Base, and the experimental data were provided by Yangzhou University, Jiangsu Province, China. The shooting equipment was a DJI Mavic 3M aerial photography drone with a shooting altitude of 25 meters. In order to solve the problem that the pixels of each image are too large and unfavorable for training, this study adopts a random cropping method, in which the original images are randomly cropped into 600 images of field ridges with a size of 512 × 512 pixels, and the dataset is divided into a training set and a validation set according to the ratio of 5:1.

This remote sensing dataset is primarily used for farmland ridge segmentation. As shown in **Figure 2**. The area of field ridges in agricultural fields is relatively small. The ridge has a regular shape in the whole, showing a thin and narrow strip, but still has complex and irregular edge texture information at the edge of the ridge. At the same time, there are tiny vacancies in the crop area of some fields, and these vacancies will directly affect the correct detection of ridges, thus, when marking the dataset, it is necessary to make accurate identification and judgment of the ridge information.

Based on the characteristics mentioned above, this study labeled the dataset using ENVI software, which has robust data processing capabilities. The software supports users with high quality data processing, analysis and applications. It can be accurately labeled for high-resolution remote sensing photos and edge complex ridge information. In this study, the fields are labeled in yellow and the crops are labeled in black to help distinguish between the ridges and the fields.

### 2.2 Experimental design

Since the connectivity of ridge shape is an important characteristic of ridges and the edges of ridges are complex and irregular, it makes it necessary to take into account the rich contextual information and long-distance feature dependencies when designing the model. For this reason, this study designs a segmentation method based on encoder-decoder architecture with strip pooling and ASPP to capture the complete shape of the ridge and clearly delineate the edges of the ridge.

#### 2.2.1 Encoder–decoder architecture

The encoder-decoder architecture used by ASPNet allows it to combine features extracted by the encoder with features upsampled on the decoder. Additionally, it enables the output result to retain the effective edge texture features while restoring to the original resolution size (Ilyas et al., 2022; Zhou et al., 2022; Ren et al., 2023).

**Figure 3:**
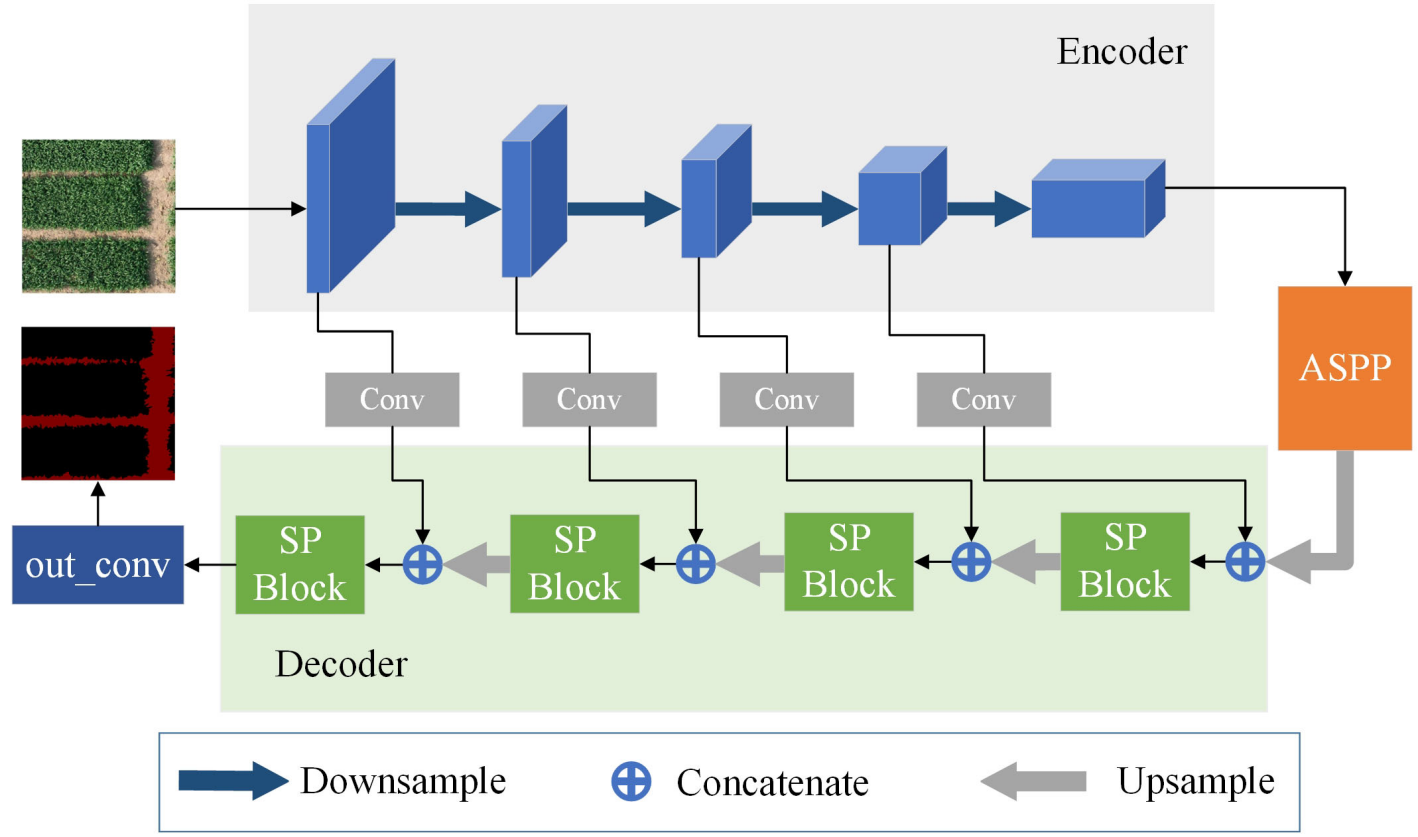
Model structure diagram.

**Figure 3** depicts the structure of the model. The encoder receives the input image initially, and the convolutional blocks in the encoder extract and output the ridge features at various scales. As the entire network is deepened, the size of the output feature maps of each block gradually decreases. In the encoder, except for the last layer of the feature map passed into the ASPP, the feature maps output from the first four convolutional blocks have two branches, one branch is used as an input to the next convolutional block, and the other is used for the feature fusion operation in the decoder. The feature maps after ASPP processing are up-sampled and used as input to the decoder. In the decoder, the up-sampled feature map will be feature fused with the output of the encoder. This feature fusion operation helps the decoder to better understand the feature information of the input image and generate more accurate predictions. Following a strip-pooling process, the fused features are then used as input for the subsequent up-sampling, and so on, repeating till the output.

#### 2.2.2 Strip pooling module

There is a high demand for the strip-shape ridge segmentation effect in the farmland ridge segmentation scene, so the model must accurately capture the complete shape of the ridge and distinguish clearly between the field’s boundary and other features. The strip pooling module in SPNet (Hou et al., 2020) is cited in this paper as serving this purpose. To acquire dependencies over long distances, strip pooling uses 1×N or N×1 strip pooling kernels. It differs from traditional pooling processes, which based on square windows to extract valid features from input images and demand a significant amount of computation to create associations at pertinent regions. However, in some application scenarios such as roads and farmland ridges that have narrow and large spans, conventional pooling is difficult to capture the remote context information of features, which causes the model to miss some of the features during processing. When the input image’s features are long and narrow, strip pooling can capture their relationship in both the horizontal and vertical directions, combine them, and establish remote dependencies throughout the entire scene. It also prevents irrelevant regions from interfering with feature learning.

**Figure 4:**
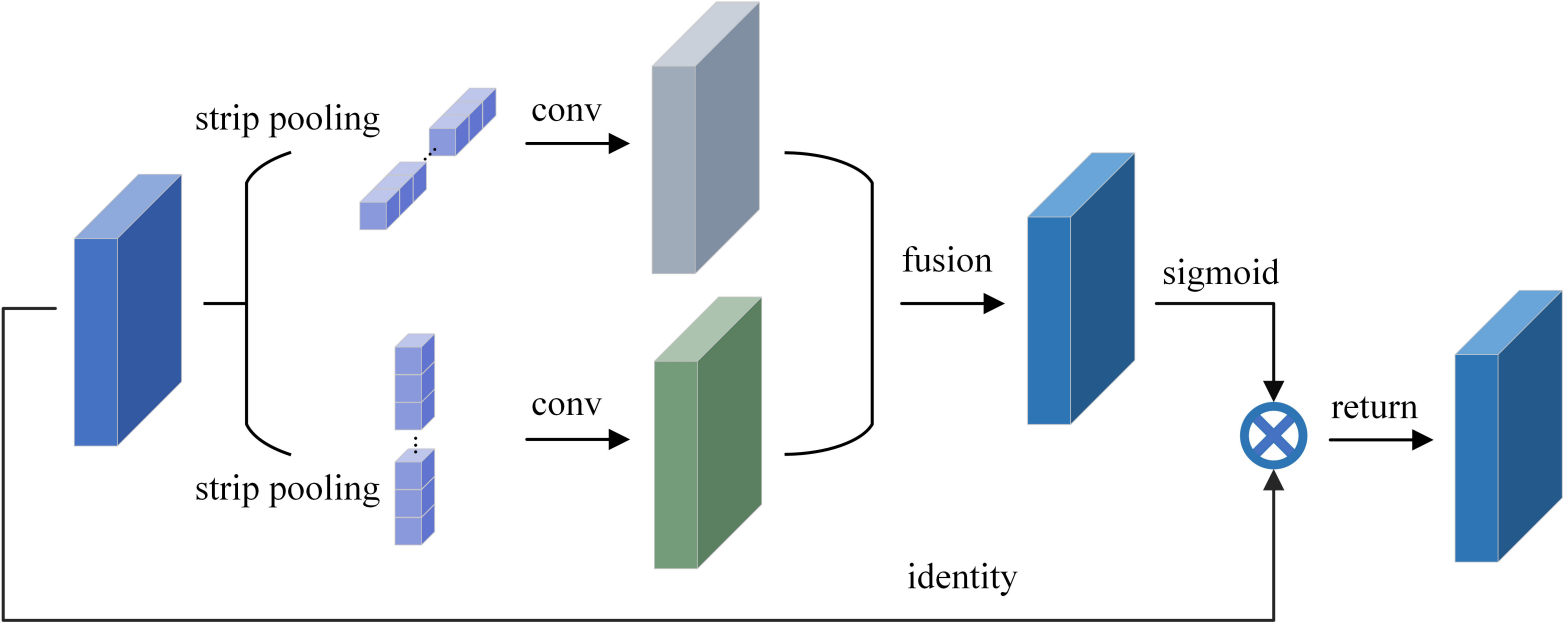
Schematic illustration of the strip pooling.

As shown in **Figure 4**, in order to average all the features in a row or column during a certain operation phase, the strip pooling module moves the strip-shaped pooling kernel in two directions, horizontal and vertical, respectively. The output of the above-mentioned pooling is then afterwards enlarged by convolution in the corresponding up-down and left-right directions. Following expansion, two H×W feature maps are created, and an H×W feature map is created by performing a pixel-by-pixel summing operation on the features corresponding to the identical positions in the two expanded feature maps. After applying a layer of convolution and sigmoid activation function processing, the output of the module is obtained by multiplying with the corresponding pixels of the original input feature map.

#### 2.2.3 ASPP

Due to the narrow shape, vast span and wide coverage of the ridge in the image, the standard convolution procedure is constrained by the receptive field and is unable to capture the rich contextual information. As a result, the atrous spatial pyramid pooling module is utilized to widen the receptive field (Chen et al., 2018), which can enhance the connectivity of ridge detection and better capture the whole contour of the ridge.

**Figure 5:**
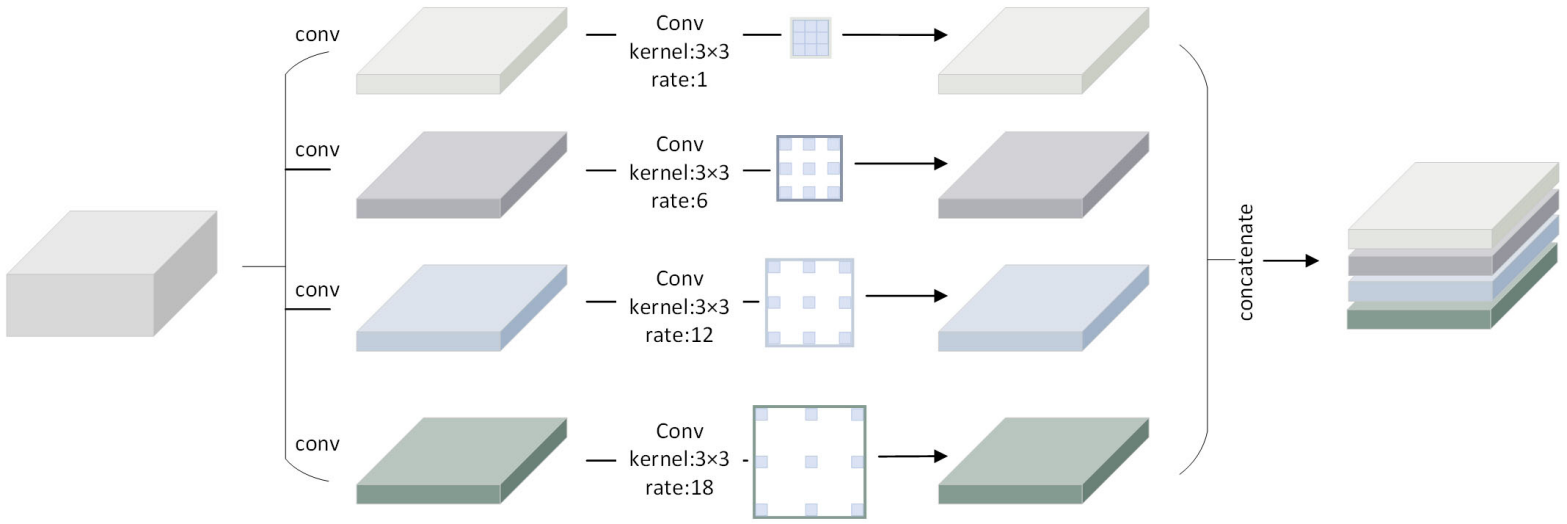
Schematic illustration of the ASPP.

To collect multi-scale contextual data, this module employs atrous convolution with various sampling rates. The output feature maps from each atrous convolution operation are then spliced and fused after the convolution operations with various sampling rates are conducted on distinct branches. Without introducing additional parameters, the expansion of the modular receptive field is achieved. The first is a 1×1 standard convolutional branch, while the following three are 3×3 convolutional branches with various sampling rates to create convolutional kernels with various receptive fields, as illustrated in **Figure 5**. In order to improve perceptual ability and semantic information, the ASPP is positioned after the encoder to process the encoder’s deepest feature output.

### 2.3 Network training and performance evaluation

A computer with a processor of Inter(R) Core(TM) i7-11700k @3.60GHz, 32G RAM, and a graphics card of NVIDIA GeForce RTX 3070 (8G RAM) was used in this study. The software environment consists of Python 3.8, CUDA 11.4, Linux 10, and PyTorch 1.8.0. This study set the starting learning rate to 0.01, the initial batch size of all the datasets to 4, the input image resolution size to 512×512, and the epoch to 100 for training the model. Additionally, the model uses SGD as the optimizer to get better training results because SGD has good randomness and simplicity in updating the learning rate, and it can have better stability throughout the model training process. This prevents the model from fitting too quickly in the early stages of training, which can result in overfitting.

The cross-entropy loss function is used in this work to quantify the discrepancy between model predictions and actual results (Zhang et al., 2021). The loss function (Equation 1) looks like this:


(1) $$loss\left(x,class\right)=-x\left[class\right]+log\left(\sum_{j}exp\left(x\left[j\right]\right)\right)\;$$

Where, x denotes the input vector and each value in the vector denotes the model predicted value. class denotes the labeled values of the different classes and a label value of 0 denotes the background and 1 denotes the ridge information.

Four quantitative criteria were employed in this study to assess the segmentation findings. The segmentation performance (Zou et al., 2022; Shunying et al., 2023) is assessed and compared using the following metrics: overall pixel accuracy (Acc), precision (Pr), recall (Re), and intersection ratio union (IoU) (Equations 2-5). The test set’s photos are averaged for Acc, Pr, Re, and IoU.


(2) $$Acc=\frac{\sum TP+\sum TN}{\sum TP+\sum TN+\sum FP+\sum FN}\times 100\%\;$$

(3) $$Pr=\frac{\sum TP}{\sum TP+\sum FP}\times 100\%\;$$

(4) $$Re=\frac{\sum TP}{\sum TP+\sum FN}\times 100\%\;$$

(5) $$IoU=\frac{\sum TP}{\sum TP+\sum FN+\sum FP}\times 100\%\;$$

In the above formula, TP is true positive, the model prediction is the positive example, and the label is the positive example; FP is false positive, the model prediction is the positive example, and the label is the negative example. FN is false negative, the model prediction is the negative example, and the label is the positive example. TN is true negative, the model predicts the negative example, and the reality is the negative example.

## 3 Results

### 3.1 Training process presentation

**Figure 6:**
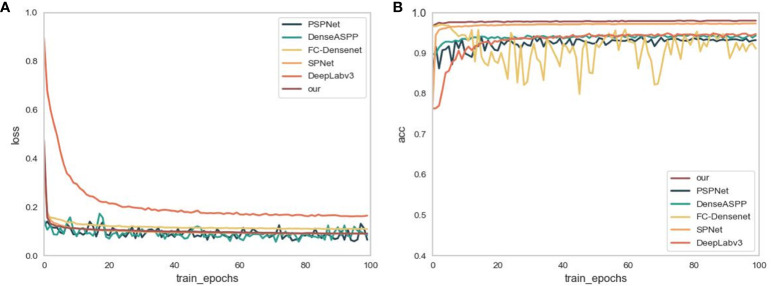
**(A)** shows the changes in loss during the training process. **(B)** is the change in accuracy during the training process.

In order to validate the effectiveness of the models proposed in this study, ASPNet is compared with DeepLabv3 (Chen et al., 2018), FC-Densenet (Jegou et al., 2017), PSPNet (Zhao et al., 2017), DenseASPP (Yang et al., 2018) and SPNet. To achieve a fair comparison, the same training dataset and validation set are used. **Figure 6** shows the variation of the loss function of the above models during the training process. In the graph, the training batch is taken as the horizontal coordinate and the corresponding loss and acc values are taken as the vertical coordinates.

As demonstrated in **Figure 6A**, the loss function values of FC-DenseNet, SPNet, DeepLabv3 and our proposed ASPNet, are high when the model is first trained. As the model is trained, the loss function values decrease, with ASPNet being the first to converge to the lowest values. While PSPNet and DenseASPP exhibit an undulating trend during training, this indicates that the model’s training is unstable and susceptible to overfitting. On the whole, the loss function value of the ASPNet tends to be stable during training rather than experiencing significant up or down swings. It indicates that the model is not easily affected by outliers, and the fitting to the noisy data is smoother and more stable, with good robustness.

**Figure 6B** shows the variation of model accuracy during training. As shown in the figure, the model recognition accuracies of the three models, SPNet, DeepLabv3, and our proposed ASPNet, show an overall upward trend during validation. While the segmentation accuracies of PSPNet and DenseASPP fluctuated significantly in the first 80 batches and gradually stabilized in the last 20 batches. The accuracy scores after stabilization, although between 0.9 and 1, are still lower than the ASPNet and SPNet accuracy values. FC-DenseNet has the most obvious oscillation trend, and combined with the gradual smoothing of its loss curve, it can be seen that the model is overfitted during the training process, ignoring some of the main features of the dataset. In summary, compared with other models, ASPNet reaches the highest accuracy the fastest during the training process and tends to stabilize with an upward trend, which shows that ASPNet can learn the feature information of ridge quickly.

### 3.2 Evaluation of segmentation

In order to verify the generalization and superiority of the proposed model, this study evaluates ASPNet and the above comparative models on a validation set. **Table 1** displays the segmentation performance scores for the various models based on the four assessment criteria Accuracy, mIoU, Precision, and Recall.

**Table 1:** Evaluation of segmentation results of different segmentation methods.

Methods	Accuracy (%)	mIoU(%)	Precision(%)	Recall(%)
DeepLabv3	94.8%	87.3%	84.4%	93.1%
FC-Densenet	97.5%	93.2%	**95.0%**	93.2%
PSPNet	95.9%	89.2%	92.0%	88.3%
DenseASPP	95.7%	89.0%	91.0%	88.9%
SPNet	97.3%	92.8%	93.6%	93.5%
ASPNet (Ours)	**98.0%**	**94.7%**	94.3%	**96.5%**

The bold values represent the highest values for each column of evaluation index.

**Figure 7:**
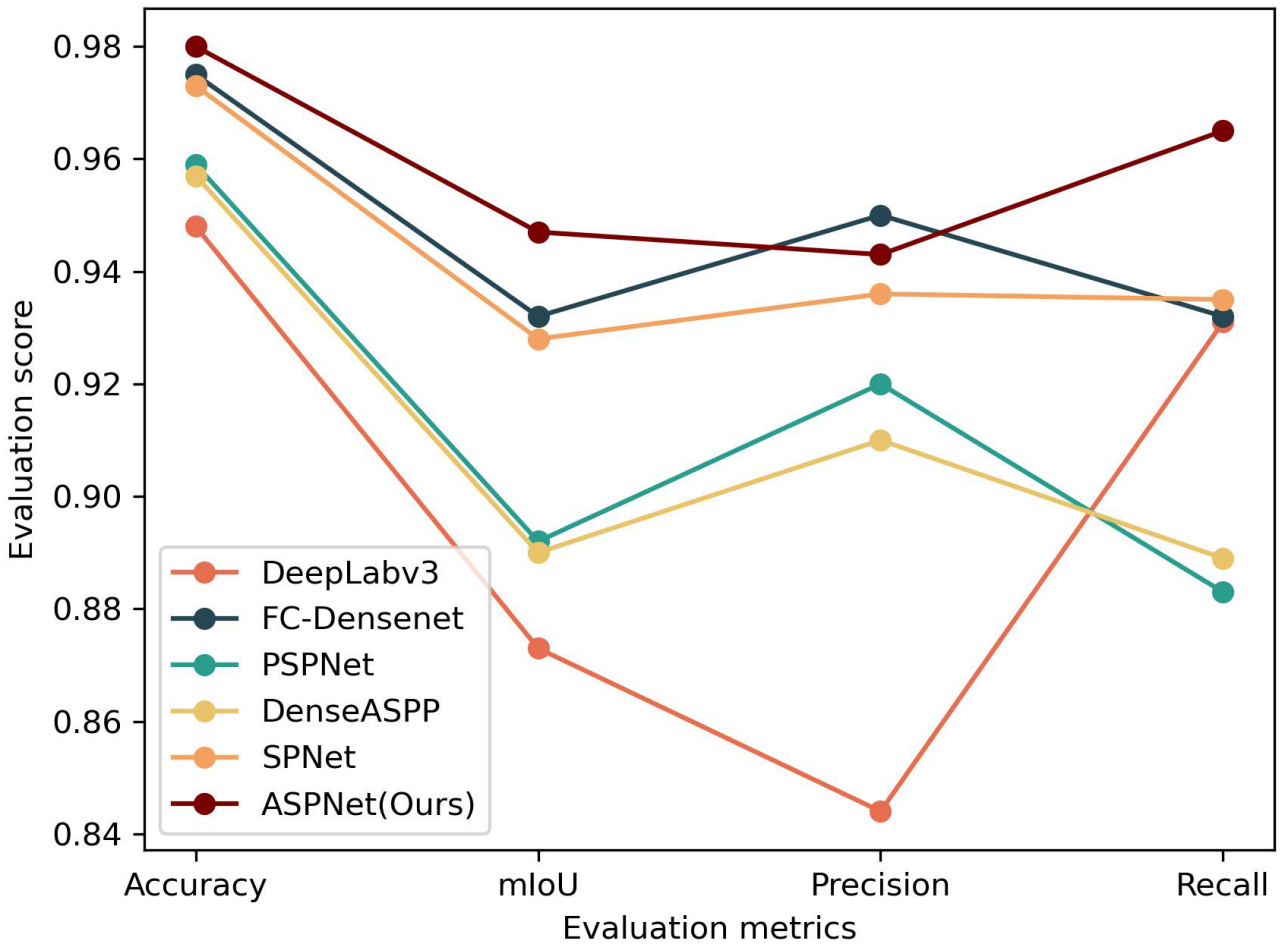
Line chart of different evaluation results.

As shown in **Table 1**, in terms of the segmentation performance of the agricultural ridge region, the proposed model ASPNet achieves the accuracy of 98.0%, the mIoU of 94.7%, the precision of 94.3%, and the recall of 96.5%. In order to get a more intuitive feel of the performance effects of the models on different evaluation metrics, this study further presents a visualization comparison of the segmentation effects of different models by line chart. Combined with the line chart in **Figure 7**, it can be seen that in terms of Accuracy, the model in this study achieved an improvement relative to DeepLabv3, FC-Densenet, PSPNet, DenseASPP, and SPNet model, with an improvement of 3.2%, 0.5%, 2.1%, 2.3%, and 0.7%, respectively. In terms of mIoU metrics, the model in this study also showed superiority, improving by 7.4%, 1.5%, 5.5%, 5.7%, and 1.9% with respect to DeepLabv3, FC-Densenet, PSPNet, DenseASPP, and SPNet model, respectively. For recall, the models in this study improved 3.4%, 3.3%, 8.2%, 7.6%, and 3% relative to DeepLabv3, FC-Densenet, PSPNet, DenseASPP, and SPNet model, respectively. Notably, the model proposed in this study scored 0.7% lower than FC-Densenet on Precision, but the error is within 1%, which is within the acceptable range.

### 3.3 Visualization results

**Figure 8:**
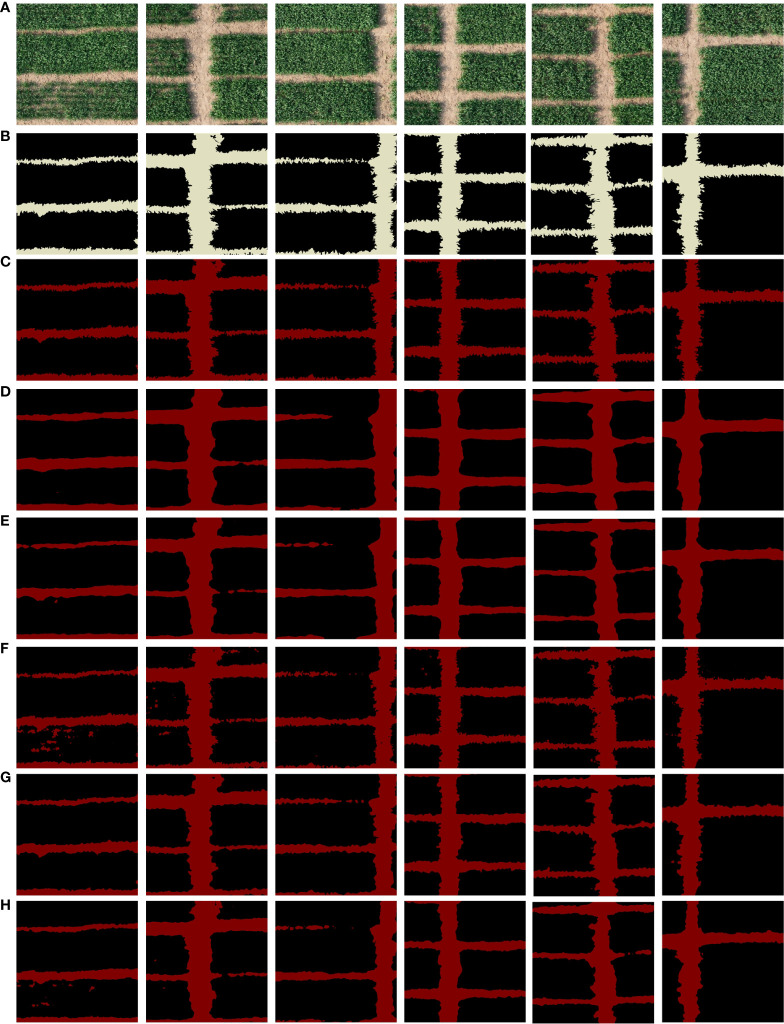
Segmentation results of different models. **(A)** Original image, **(B)** ground truth, and **(C)** visual results of our proposed, and **(D)** for the visual results of DeepLabv3, **(E)** visual results of PSPNet, **(F)** visual results of FC-DenseNet, **(G)** visual results of SPNet, and **(H)** visual results of DenseASPP.

Results of segmentation for ASPNet and comparative models are provided in this paper. As seen in **Figure 8**. Among them, the group of **Figure 8A** shows the input images used to test the segmentation effect, and all of these farmland images have different ridge distributions, but have obvious horizontal or vertical strip-like features. Some of the ridges in the images have distinctive features and cover a large area, while others are thin and narrow between crops. In addition, there are some tiny vacancies in two crop fields, which can be used as interference information in the ridge prediction segmentation process. This provides an intuitive reference basis for observing the segmentation effect in this study. The areas in the label and segmentation result map, with the exception of the ridge, are painted in black as the background in order to make the segmentation effect more obvious. In **Figure 8**, group (B) shows the ridge data labels, group (C) shows the segmentation results of ASPNet, the model proposed in this study, and group (D)-(H) shows the segmentation results of the comparison model.

According to the segmentation results of different models, in the segmentation results of groups (D) and (E), DeepLabv3 and PSPNet’s prediction of narrow field ridge information between crops is incomplete. Some of the ridge information is therefore missing in the segmentation map since they are unable to accurately detect the ridge information in the image. As demonstrated in group (F), FC-DenseNet improperly segments the interference information of fine vacancies on the field into ridge categories, as well as the segmentation outcomes of DenseaASPP in group (H). It also fails to accurately distinguish parts of the fine and narrow ridge information. This shows that the model only picks up on a limited number of characteristics during training, and that it struggles to pick up on the spatial aspects characteristics of the ridges. As a result, the model’s judgment of the interference information during the detection segmentation phase is not accurate enough, which leads to low model robustness. The model is not fine enough to segment the ridge boundary, as shown by the SPNet segmentation results in group (G). And the effect of the model proposed in this study is shown in (C), the segmentation results are very similar to the markers in the labels, the model is able to exclude the interference of small vacancies in the agricultural field blocks, even the shape of thin and narrow ridges can be recognized and marked by the model, and it has the ability to capture the complex boundaries of the ridges.

### 3.4 Results of Large-farmland

**Figure 9:**
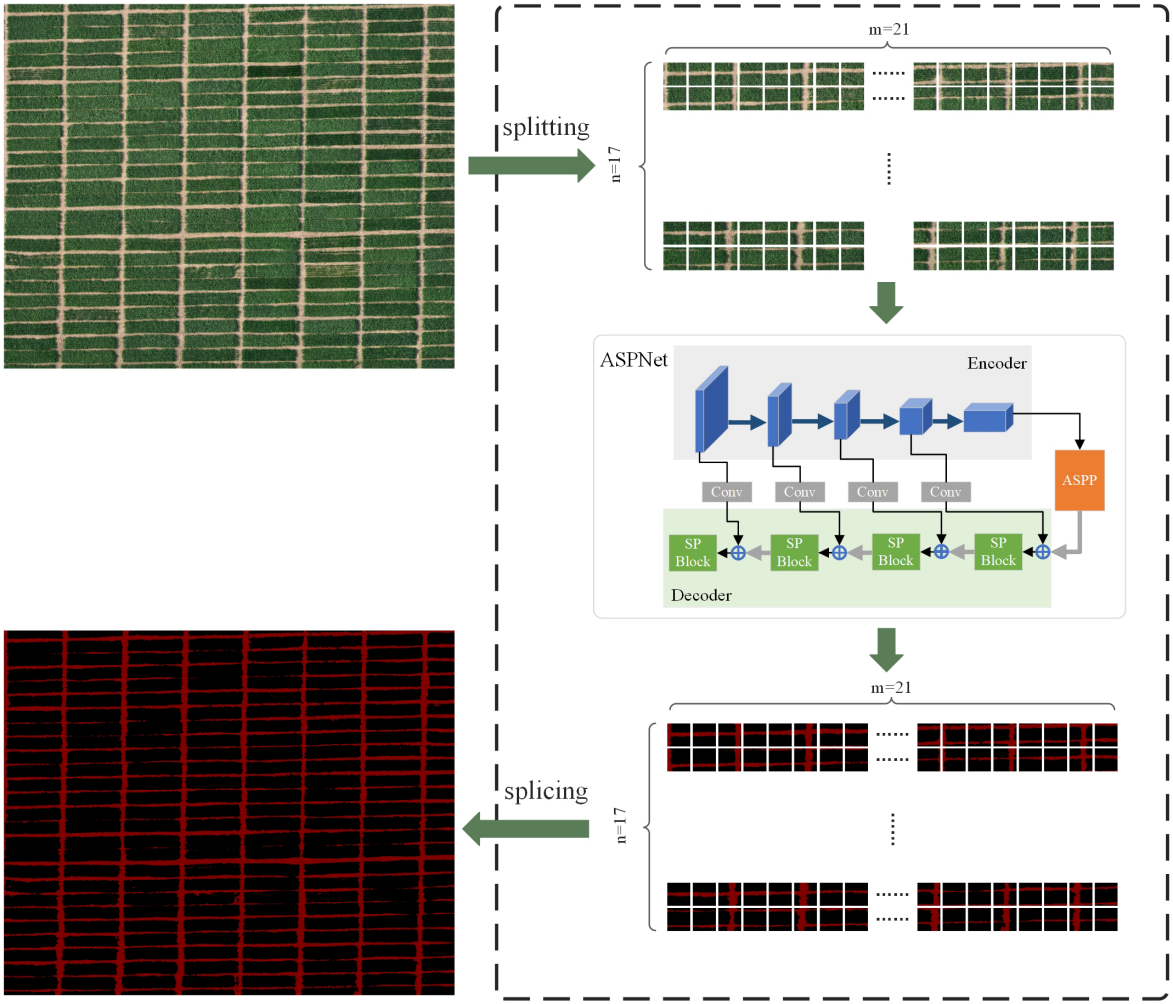
Operational flow of large-area farmland segmentation.

Considering that in practical application scenarios, ridge segmentation is required for large-area farmland. In this study, based on the above small-resolution ridge segmentation process, the farmland image with a resolution of 10752×8704 is segmented to realize the acquisition of ridge distribution information for farmland covering an area of about 7000 square meters. The ASPNet proposed in this study is primarily for the segmentation of 512×512 resolution images due to assure the processing speed and accuracy of the model. In order to be able to adapt to the needs of ridge segmentation in a larger area, this study has considered the reliability and applicability of the actual segmentation results in designing the segmentation task for large-area farmland. The whole segmentation process firstly splits the input image into n×m small blocks with the resolution of 512×512; then inputs each small block into the model of this study for segmentation; and finally splices the n×m small blocks together to create the segmentation result of the large-area farmland. This end-to-end farmland ridge segmentation method makes the processing flow simpler while guaranteeing accurate segmentation. The segmentation process is shown in **Figure 9**.

**Figure 10:**
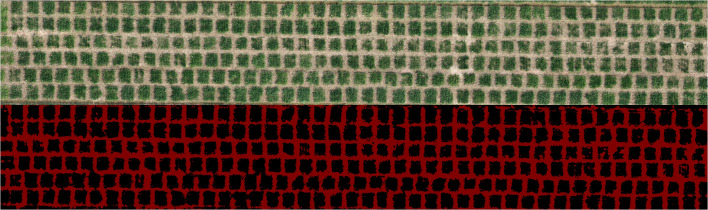
Result of ridge segmentation in another experimental field.

During the above data processing, it took 26.115 seconds to cut the large farmland image into small resolution images. All small resolution images were transferred into the model for processing in 24.148 seconds, with an average processing time of 0.047 seconds per small resolution image. 4.729 seconds were used in stitching into a large agricultural field ridge segmentation map. It can be seen that the model proposed in this study can extract ridge information accurately and efficiently from farmland images of about 7000 square meters in one minute. In addition, the ridge segmentation was also tested in another experimental field in this study. The experimental field is located in Wanfu Experimental Base in Yangzhou City, Jiangsu Province, covering an area of 1,314 square meters with a shooting height of 20 meters. The segmentation results are shown in **Figure 10**.

## 4 Discussion

A segmentation model that can learn accurately on small datasets must be designed in order for the model to accurately and efficiently identify the ridge information because the use of remote sensing equipment carried by unmanned aerial vehicles (UAVs) to acquire remote sensing data of field ridges necessitates consideration of geographic and time scale issues as well as seasonal variations. The convolutional neural network is one of the most utilized machine learning models today that can accurately perform farmland segmentation. (Potlapally et al., 2019), in order to advance the automated analysis of remote sensing data for land use, used Mask R-CNN to classify and segment farmland of various crop types, which is a model that adds pixel-level segmentation of each target instance on the basis of target detection. Additionally, the encoder-decoder architecture provides a method for pixel-by-pixel segmentation that is efficient. To help the model better capture detail information and increase segmentation accuracy, it fuses the low-level features of the encoder with the high-level features of the decoder. (Wang et al., 2023) inspired by the encoder-decoder architecture, proposed a multi-task deformable UNet combinatorial enhancement network based on UNet, which consists of a shared universal encoder part and three independent decoder parts, to realize high-precision segmentation of farmland boundaries, effectively preserving the edge texture information. Therefore, this study adopts the encoder-decoder architecture to accurately and efficiently realize the pixel-by-pixel segmentation of the whole image. In the encoder stage, conventional convolution is used for local feature extraction. The features output from the encoder are then mixed with those from the decoder, and the output is up-sampled to return to the original input resolution size. The issue of pixel space information loss can be effectively dealt with by making good use of the shallow texture information and deep semantic information of the feature map.

However, the above improved models based on Mask R-CNN and U-Net lack the extraction of information between different receptive fields during feature extraction. The appearance of atrous convolution can help the model to establish the connection between different receptive fields in the feature map. In the study of farmland segmentation by (Du et al., 2019), the DeepLabv3+ model with atrous convolution is used to extract and map the distribution of the crops in order to accurately describe the small and irregular fields in the farmland. It is demonstrated by experimental comparisons that DeepLabv3+ with atrous convolution is effective in obtaining the information about the distribution of the farmland. In order to increase the precision of farmland segmentation, (Sun et al., 2022) suggested a DeepLabv3+ based deep edge enhanced semantic segmentation network. While keeping the atrous convolution, they added supplementary labels to strengthen the model’s learning capabilities and increase the performance of ridge and cropland recognition. Considering the effectiveness of atrous convolution, this study adds ASPP module in the middle of encoder and decoder. By using the atrous convolution in ASPP to widen the receptive field, the model is better able to capture the overall contour of the ridge.

The pooling kernel in traditional convolutional neural networks is typically square and only takes into account local contextual information, neglecting the interdependence of distant features. A novel pooling processes called strip pooling is presented in the paper by (Hou et al., 2020). To represent remote dependencies, it utilizes a long and narrow pooling kernel. By contrasting strip pooling with conventional spatial pooling, the study highlights the extraction capability of strip pooling on banded features (Mei et al., 2021). (Zhang et al., 2021) applied strip pooling module in ridge segmentation to capture the effective information of ridges. By contrasting it with well-known semantic segmentation models, the study showed the strip pooling module’s dependability in ridge segmentation settings. Therefore, considering that stripes are one of the main features of ridges in ridge segmentation scenarios, this study adds the strip pooling module to the decoder to enhance the extraction of strip features of ridges and to delineate the edges of ridges from other elements.

In summary, we propose a ridge segmentation method based on an encoder-decoder architecture, which incorporates an ASPP module after the encoder and the strip pooling modules in the decoder. The experimental results demonstrate that this method has good segmentation effect in ridge segmentation scenarios.

## 5 Conclusion

In this study, a segmentation method for farmland ridges is proposed for the characteristics of narrow shape, complex and irregular edges. Firstly, in order to provide effective data support, the ridge dataset from remote sensing images of agricultural fields was collected and produced in this study. Then, a segmentation method based on encoder-decoder architecture with strip pooling and ASPP is designed to achieve accurate segmentation of ridge information in agricultural fields. Finally, the model is evaluated based on the validation set, and the evaluation results show that the model outperforms the comparison model in terms of ridge segmentation effect, in which the accuracy reaches 98% and the mIoU score is 94.6%. In practical application scenarios, it can quickly realize the accurate segmentation of ridges in large-area farmland images and output the segmentation results of complete farmland. The method can not only accurately segment the shape information and fine edge information of field ridges, but also avoid the interference caused by the tiny vacancies between farm fields. In addition, it can promote the optimal use of resources by farmers, thus improving productivity as well as reducing environmental impact and accelerating the realization of unmanned farmland management.

Since producing a dataset for segmentation of agricultural field ridges is labor-intensive and time-consuming, in future research, we expect to combine semi-supervised and unsupervised learning approaches to achieve segmentation of agricultural field ridges using a small number of datasets. And the model can be lightweighted so that make it more easily applicable to edge devices in farmland for completing mechanical work.

## Data availability statement

The original contributions presented in the study are included in the article/supplementary material. Further inquiries can be directed to the corresponding author.

## Author contributions

QH: Conceptualization, Formal analysis, Investigation, Methodology, Validation, Visualization, Writing – review & editing. YZ: Conceptualization, Formal analysis, Investigation, Methodology, Software, Validation, Visualization, Writing – original draft, Writing – review & editing. WL: Software, Writing – review & editing. TR: Software, Writing – review & editing. CS: Supervision, Writing – review & editing. ZLu: Supervision, Writing – review & editing. YY: Supervision, Writing – review & editing. RD: Supervision, Writing – review & editing. JQ: Supervision, Writing – review & editing. CT: Supervision, Writing – review & editing.
